# Secondary Adrenal Insufficiency After COVID-19 Diagnosed by Insulin Tolerance Test and Corticotropin-Releasing Hormone Test

**DOI:** 10.7759/cureus.23021

**Published:** 2022-03-10

**Authors:** Kenya Hamazaki, Tomoko Nishigaki, Naoki Kuramoto, Koji Oh, Hiroki Konishi

**Affiliations:** 1 General Internal Medicine, Kobe City Medical Center West Hospital, Kobe, JPN; 2 Diabetes and Endocrinology, Kobe City Medical Center West Hospital, Kobe, JPN

**Keywords:** hypothalamic–pituitary–adrenal axis, post-covid-19 conditions, long covid, insulin tolerance test, corticotropin-releasing hormone test, secondary adrenal insufficiency, central adrenal insufficiency, adrenocorticotropic hormone deficiency, severe acute respiratory syndrome coronavirus 2, coronavirus disease 2019

## Abstract

Coronavirus disease 2019 (COVID-19) can affect multiple organs and systems, including the endocrine system. Its symptoms can last for months, resulting in post-COVID-19 conditions, among others. A small number of patients have central adrenal insufficiency (CAI) months after recovery from COVID-19; nevertheless, its pathogenesis has not been fully elucidated. The insulin tolerance test (ITT) is a gold standard test assessing the hypothalamic-pituitary-adrenal axis, and the corticotropin-releasing hormone (CRH) test is useful for differentiating CAI into secondary (pituitary) and tertiary (hypothalamic) adrenal insufficiency. We present a case of new-onset CAI in a young female patient who had no medical history other than COVID-19. Adrenocorticotropin hormone and cortisol responded poorly to both insulin-induced hypoglycemia and CRH administration. These findings suggest that the pituitary gland may be the primary site of hypothalamic-pituitary-adrenal dysfunction, although magnetic resonance imaging of the pituitary gland was unremarkable. To our knowledge, this is possibly the first and only case report of new-onset secondary adrenal insufficiency after recovery from COVID-19 in which the ITT and the CRH test were performed and highly suggestive for the pathogenesis of not only post-COVID-19 CAI but also post-COVID-19 conditions.

## Introduction

To enter host cells, the severe acute respiratory syndrome coronavirus 2 (SARS-CoV-2) utilizes angiotensin-converting enzyme 2 (ACE2) as an entry receptor. Coronavirus disease 2019 (COVID-19) may be able to target tissues that express ACE2 receptors. Many tissues have been found to have ACE2 receptors, including the intestine, testis, kidneys, heart, thyroid, adipose tissue, lungs, colon, liver, bladder, adrenal gland, pituitary gland, and hypothalamus [1,2]. Urhan et al. (2022) discovered that COVID-19 can occasionally affect hypothalamus or pituitary functions, particularly the hypothalamic-pituitary-adrenal (HPA) and growth hormone (GH) axes [3]. However, there are few case reports of symptomatic central adrenal insufficiency (CAI) after recovery from COVID-19.

CAI is characterized by impaired adrenocorticotropin hormone (ACTH) secretion because of a disease or injury to the hypothalamus or the pituitary gland leading to reduced cortisol production. CAI can be caused by interference with ACTH secretion by the pituitary (secondary) or interference with CRH secretion by the hypothalamus (tertiary). The CRH test can be used to differentiate between hypothalamic and pituitary causes. Patients with pituitary failure do not respond well to CRH administration, whereas those with the hypothalamic disease usually do. Poor response of ACTH to the insulin tolerance test (ITT) but a good response to CRH suggests tertiary adrenal insufficiency (AI). Although the ITT and the CRH test are helpful in the differential diagnosis of CAI, because the differentiation between secondary and tertiary AI is seldom important from a therapeutic standpoint, they are not used in clinical practice very often. Thus, there have been no reports of cases of post-COVID-19 CAI in which the ITT and the CRH test were performed until now. The case we report may be suggestive in considering the pathogenesis of post-COVID-19 CAI.

## Case presentation

A 23-year-old female presented to our hospital’s outpatient clinic with low-grade fever, weakness, loss of appetite, and weight loss. Her menstrual cycle had also been delayed by two weeks. She denied experiencing any of the following symptoms: vomiting, abdominal pain, diarrhea, or any other. Her symptoms began two months before her admission, and she lost 10 kg of body weight during that time. She had no other medical history besides COVID-19 and no history of pregnancy. One month before the onset of her symptoms, which was confirmed by a positive reverse transcription-polymerase chain reaction test for SARS-CoV-2. She described her symptoms at the time as fever, anosmia, and ageusia, which improved in several days, two weeks, and three weeks, respectively. She had received no treatment for COVID-19, including glucocorticoid administration. She had been isolated and treated at home due to the mild disease severity. It was three weeks after her second dose of BNT162b2 SARS-CoV-2 vaccine (Pfizer, Inc; Philadelphia, Pennsylvania) that she contracted COVID-19. Incidentally, the virus she had been infected with was probably the Delta variant, which the World Health Organization classified as a variant of concern because it accounted for nearly all of the SARS-CoV-2 infections at that time in Japan.

**Figure 1:**
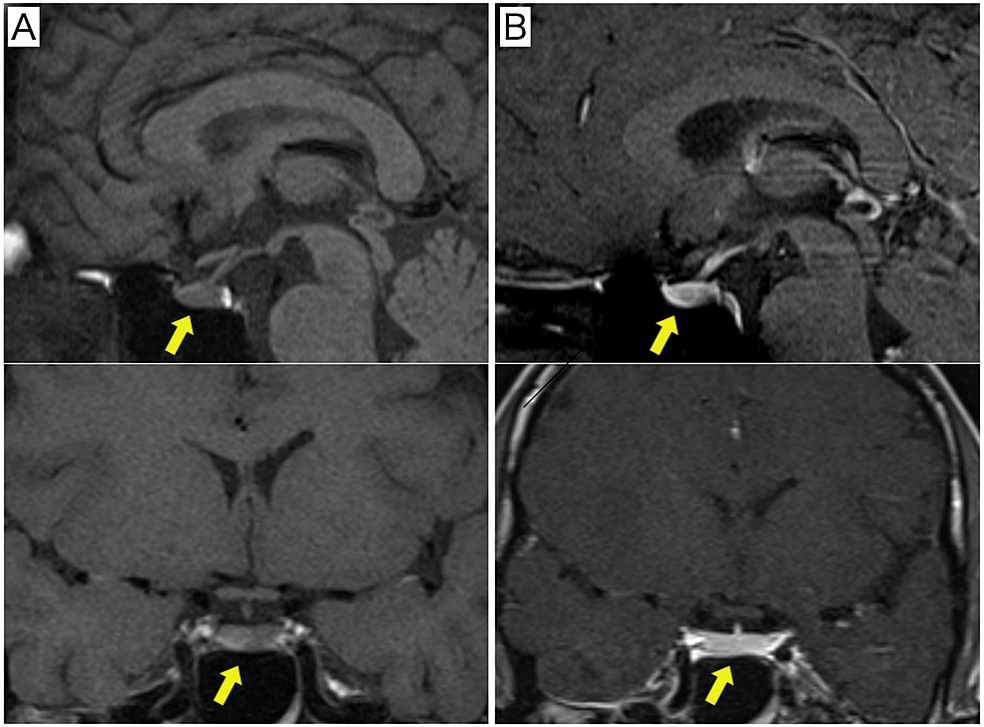
Magnetic resonance imaging of the hypothalamic–pituitary axis. Upper: sagittal plane. Lower: coronal plane. (A) T1-weighted image. (B) Gadolinium-enhanced T1-weighted image. Yellow arrows indicate the normal pituitary gland.

Her physical examination was unremarkable. The serum sodium and potassium levels, eosinophil count, fasting blood glucose level, and urinary density were in normal ranges. At 09:00, the serum cortisol level was 8.2 µg/dL, and the ACTH level was 4.9 pg/mL. Table 1 shows the laboratory workup. We suspected CAI on the basis of her laboratory examination findings, although magnetic resonance imaging (MRI) of the pituitary gland was negative for any pituitary mass or acute hemorrhage (Figure 1).

**Table 1:** Summary of the laboratory workup.

Laboratory test	Results	Reference ranges
White blood cell	7,070/μL	3,900–9,800/μL
Neutrophil	64.7%	40.0–75.0%
Lymphocyte	28.9%	18.0-49.0%
Monocyte	3.4%	2.0–10.0%
Eosinophil	2.4%	0.0–8.0%
Basophil	0.6%	0.0–2.0%
Red blood cell	465 × 10^3^/μL	350–510 × 10^3^/μL
Hemoglobin	12.9 g/dL	11.1–15.1 g/dL
Hematocrit	39.3%	33.5–45.1%
Platelet count	25.8 × 10^4^/μL	13.0–37.0 × 10^4^/μL
Total protein	7.72 g/dL	6.30–8.30 g/dL
Albumin	4.37 g/dL	3.80–5.10 g/dL
Aspartate aminotransferase	18 U/L	9–35 U/L
Alanine aminotransferase	11 U/L	5–36 U/L
Lactate dehydrogenase	146 U/L	124–222 U/L
Alkaline phosphatase	175 U/L	110–370 U/L
Blood urea nitrogen	13 mg/dL	6–22 mg/dL
Creatinine	0.76 mg/dL	0.47–0.79 mg/dL
Na	139 mEq/L	137–144 mEq/L
K	4.6 mEq/L	3.6–4.8 mEq/L
Cl	103 mEq/L	101–108 mEq/L
C-reactive protein	<0.10 mg/dL	0.0–0.5 mg/dL
Blood glucose	86 mg/dL	70–109 mg/dL
Thyroid-stimulating hormone	2.33 uIU/mL	0.50–5.00 uIU
Free thyroxine	1.1 ng/dL	0.90–1.70 ng/dL
Cortisol	8.2 μg/dL	3.7–19.4 μg/dL
Adrenocorticotropin hormone	4.9 pg/mL	7.2–63.3 pg/mL
Growth hormone	1.49 ng/mL	0.13–9.88 ng/mL
Insulin-like growth factor-1	226 ng/mL	155–397 ng/mL
Prolactin	17.03 ng/mL	6.12–30.54 ng/mL
Follicle-stimulating hormone	2.20 mIU/ml	1.47–8.49 mIU/mL
Luteinizing hormone	6.37 mIU/mL	1.13–14.22 mIU/mL
Estradiol	22 pg/mL	78–252 pg/mL
Progesterone	3.9 ng/mL	8.5–21.9 ng/mL
24-hour urinary free cortisol	50.1 μg/day	11.2–80.3 μg/day

**Figure 2:**
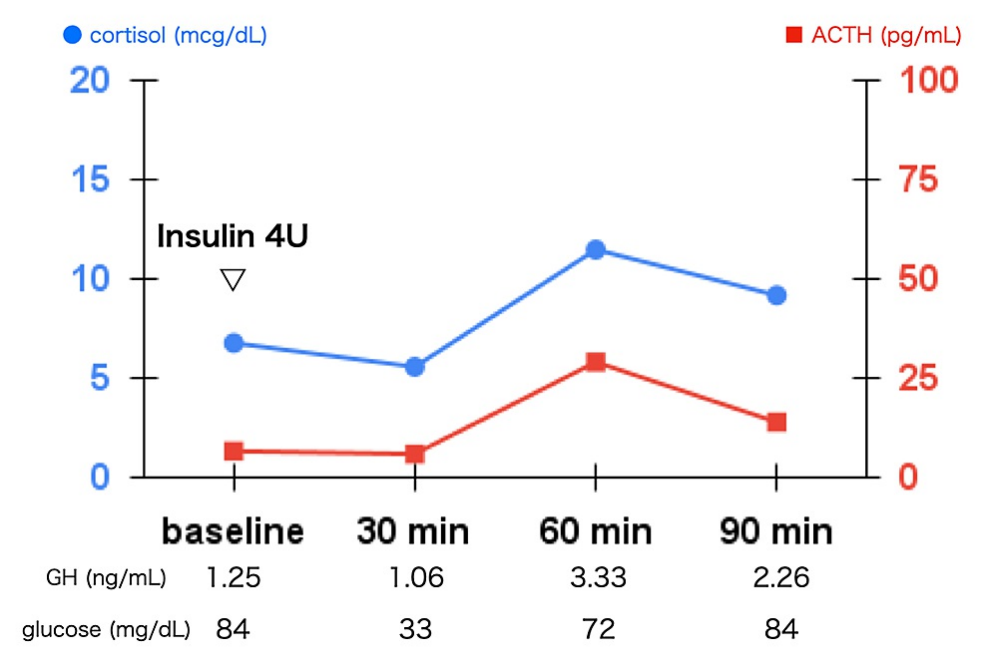
Insulin tolerance test. Adequate hypoglycemia, defined as 35 mg/dL or less, was achieved by insulin administration; however, the responses of ACTH and cortisol to insulin-induced hypoglycemia were inadequate. Although the response of GH was slightly poor, there was considered to be no severe secretory deficiency. ACTH: adrenocorticotropic hormone; GH: growth hormone

**Figure 3:**
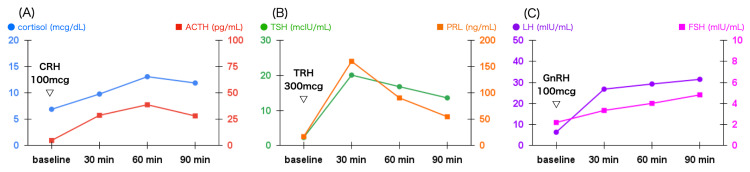
Combined pituitary stimulation test (A) CRH test. (B) TRH test. (C) GnRH test. The combined pituitary stimulation test revealed that the ACTH reactivity to CRH was selectively impaired, while the other pituitary hormones responded appropriately. Cortisol levels also increased poorly after CRH administration. CRH: corticotropin-releasing hormone; TRH: thyrotropin-releasing hormone; GnRH: gonadotropin-releasing hormone; ACTH: adrenocorticotropic hormone; FSH: follicle-stimulating hormone; LH: luteinizing hormone; PRL: prolactin; TSH: thyroid-stimulating hormone

ACTH and cortisol levels increased insufficiently during the ITT (Figure 2). ACTH levels were found to be 6.8, 6.1, 29.2, and 14.1 at 0, 30, 60, and 90 minutes, respectively. Cortisol levels were found to be 6.8, 5.6, 11.5, and 9.2 at 0, 30, 60, and 90 minutes, respectively. To evaluate the responsiveness of the pituitary gland to CRH and other hypothalamic hormones, we performed the combined pituitary stimulation test by CRH, thyrotropin-releasing hormone (TRH), and gonadotropin-releasing hormone (GnRH) (Figure 3). The CRH test showed an inadequate increase in ACTH and cortisol levels. ACTH levels were found to be 4.9, 28.8, 38.8, and 28.2 at 0, 30, 60, and 90 minutes, respectively. Cortisol levels were found to be 6.9, 9.8, 13.1, and 11.9 at 0, 30, 60, and 90 minutes, respectively. The responsiveness of other pituitary hormones was in the normal range.

We diagnosed her with secondary AI based on the results of the CRH test. She was started on 10 mg of hydrocortisone daily, and her symptoms of low-grade fever, weakness, loss of appetite, weight loss, and metalepsis improved.

## Discussion

COVID-19 has been spreading globally since it was reported in Wuhan, China, in December 2019. Knowledge regarding COVID-19 has been accumulated worldwide, and methods for diagnosis, treatment, and prevention have been established. In this context, it has been discovered that some patients who are affected and recovered from COVID-19 develop a series of symptoms, which poses a new challenge. In a systematic review, the median percentage of patients experiencing at least one persistent symptom, such as fatigue and shortness of breath, amounted to 72.5% [4]. According to another systematic review, more than half of COVID-19 survivors experienced at least one persistent symptom six months after recovery [5]. They are referred to as post-COVID-19 conditions, long COVID, long-haul COVID, and post-acute sequelae of COVID-19; however, their pathogenesis remains unclear.

A review revealed that many patients after COVID-19 recovery suffered from symptoms such as fatigue (58%), nausea (16%), depression (12%), weight loss (12%), and intermittent fever (11%) [6]. It has been reported that Japanese patients also suffer from similar symptoms [7]. Chronic fatigue is the most frequent symptom after recovery from COVID-19 in Japan, especially in females [8]. Incidentally, AI is also associated with these symptoms.

Is it possible that AI is causing some of these symptoms?

COVID-19 has been linked to various organ and system dysfunctions, including the endocrine system. Several studies have shown that this virus is present in the central nervous system of SARS-CoV-2-infected people [9]. Because of its high expression of the ACE2 receptor and transmembrane protease serine 2 [10], the hypothalamus is an important target of COVID-19. Although some data on the hypothalamus/pituitary function during acute infection are available [11,12], hypothalamus/pituitary function after recovery from COVID-19 has not been fully investigated; nevertheless, a recent study revealed that low-dose ACTH stimulation tests showed an insufficient cortisol response among 16.2% of patients with a history of COVID-19 at least three months ago, and 9.3% of patients showed an inadequate response to glucagon stimulation tests (GST) [3]. The GST stimulates both ACTH and GH secretion like the ITT and can be a good alternative to the ITT because of the dangers posed by the ITT, whereas it has yet to be considered the gold standard test [13]. From this aspect, our case, in which the ITT and the CRH test were performed, may be valuable.

Approximately 10% of patients may have CAI three months after recovering from COVID-19, even though the majority of their CAI is asymptomatic [3]. By contrast, there are few case reports of symptomatic CAI after recovery from COVID-19 [14]. There may be no small number of CAI patients among those who are thought to have post-COVID-19 conditions, who are simply unrecognized. It is often mentioned that post-COVID fatigue necessitates ruling out common causes such as anemia, hyperglycemia, electrolyte imbalance, and hypothyroidism, but it is less often said that CAI should be attended to [15].

Our patient was diagnosed as having secondary AI on the basis of inadequate cortisol and ACTH secretion responses on the ITT and the CRH test. The ITT is a gold standard test to assess the integrity of the entire HPA axis, and the GST can also be used for the same purpose [16], although they are not useful for distinguishing between secondary and tertiary AI. In our case, an addition of the CRH test successfully differentiated secondary AI from CAI, which is the first time this has been done. In the above-mentioned case, MRI revealed a significantly thinner pituitary gland, although radiological records performed two years earlier for another reason were normal [14]. Conversely, there were no abnormalities in the pituitary imaging findings in our case, which is also thought to be suggestive in considering the pathogenesis of post-COVID-19 CAI.

The patient’s symptoms improved quickly after starting hydrocortisone replacement therapy, but how long should the replacement therapy be continued? As it has not been long since we started the treatment, we have not yet performed stimulation tests again. In a study of 61 patients three months after recovering from SARS in Singapore, 39% experienced transient HPA axis dysfunction with hypocortisolism that resolved within a year [17]. Although this was a SARS study rather than a COVID-19 study, SARS has many similarities with COVID-19, and the clinical characteristics of SARS are very helpful in understanding the pathogenesis of COVID-19. SARS-CoV, as well as SARS-CoV-2, also utilizes ACE2 as an entry receptor [18] and causes severe damage to multiple organs, including the lungs and microvessels [19]. A study of endocrine cells in the anterior lobe of the pituitary gland from autopsies of five SARS patients found that immunohistochemical examination revealed a marked decrease in both the number of positive cells and the staining intensity of immunoreactivity for GH, thyroid-stimulating hormone, and ACTH [20]. These remain useful references for understanding the time course and pathogenesis of post-COVID-19 CAI.

CAI is common and may be one of the etiologies of post-COVID-19 symptoms. The impact of these dysfunctions on post-COVID-19 conditions, their recovery, and follow-up requires further investigation.

## Conclusions

To the best of our knowledge, this is the first time the ITT and the CRH test have been performed on a post-COVID-19 CAI patient. Symptoms such as fatigue, nausea, depression, weight loss, and fever are common in post-COVID-19 patients; however, they are rarely examined in detail. CAI is not uncommon in post-COVID-19 patients. To manage these patients and improve their outcomes, close attention is required.
